# Case report: Intraoperative thrombosis cardiac arrest in extended right hepatectomy involving use of local haemostatic agent in intraoperative cell salvage (ICS) and administration of recombinant activated factor VII (rFVIIa)

**DOI:** 10.1016/j.ijscr.2019.02.042

**Published:** 2019-03-06

**Authors:** Lee S. Kyang, Andrew Howard, Nayef A. Alzahrani, David L. Morris

**Affiliations:** a Department of Surgery, St George Hospital, University of New South Wales, Sydney, New South Wales, Australia; b Department of Anaesthesia, St George Hospital, Sydney, New South Wales, Australia; c College of Medicine, Al-Imam Mohammad Ibn Saud Islamic University (IMSIU), Riyadh, Saudi Arabia

**Keywords:** Mortality, Recombinant activated factor VII, Intraoperative cell salvage, Thrombosis, Liver resection, rFVIIa

## Abstract

**Introduction:**

In modern surgical era, local haemostatic agents and blood components such as recombinant activated factor VII (rFVIIa) have expanded surgeons’ armamentarium in controlling “surgical” and “nonsurgical bleeding”. We report a case of intraoperative thrombosis and cardiac arrest involving use of local haemostatic agent in intraoperative cell salvage and rFVIIa administration in extended right hepatectomy.

**Presentation of case:**

A 46-year-old lady underwent extended right hepatectomy using cardiopulmonary bypass (CPB) and autotransfusion with ICS for metastatic gastrointestinal stromal tumour. She became extremely coagulopathic following weaning of CPB despite an array of fluid and blood products replacements. Decision to administer rFVIIa as a measure to arrest bleeding was unsuccessful. Extensive systemic thrombosis occurred which resulted in cardiac arrest and mortality.

**Discussion:**

The thromboembolic event was unclear but likely multifactorial. Two important hypotheses were the administration of rFVIIa and use of local haemostatic agent in ICS.

**Conclusion:**

Reported incidence of thromboembolism with use of rFVIIa in refractory bleeding is variable. More randomised controlled trials are needed to ascertain the efficacy and safety profile of the haemostatic agent. At present, off-label use of rFVIIa should be guided by the risk:benefit profile on a case-to-case basis. The authors also feel strongly against the use of local haemostatic gel in conjunction with ICS due to potential systemic circulation of the thrombin.

## Highlights

• Intractable intraoperative haemorrhage is a result of both surgical and coagulopathic (nonsurgical) components.
• There is increasing off-label use of rFVIIa for ceasing refractory bleeding aside from its application in patient with haemophilia.
• rFVIIa use may be associated with increased thromboembolic events according to some literature.
• The use of topical haemostatic agent in conjunction with ICS may potentially lead to systemic clot formation upon re-infusion of the chemical.
• Avoid use of cell saver suction while the surgical field is contaminated with topical clotting factors before irrigation with 0.9% sodium chloride.

## 1 Background

In modern surgical era, local haemostatic agents and blood components such as recombinant activated factor VII (rFVIIa) have expanded surgeons’ armamentarium in controlling “surgical” and “nonsurgical bleeding”. This paper reported a case of intraoperative thrombosis and cardiac arrest involving use of local haemostatic agent in intraoperative cell salvage and rFVIIa administration in extended right hepatectomy. This report was written in accordance with SCARE guideline [1].

## 2 Case report

**Fig. 1:**
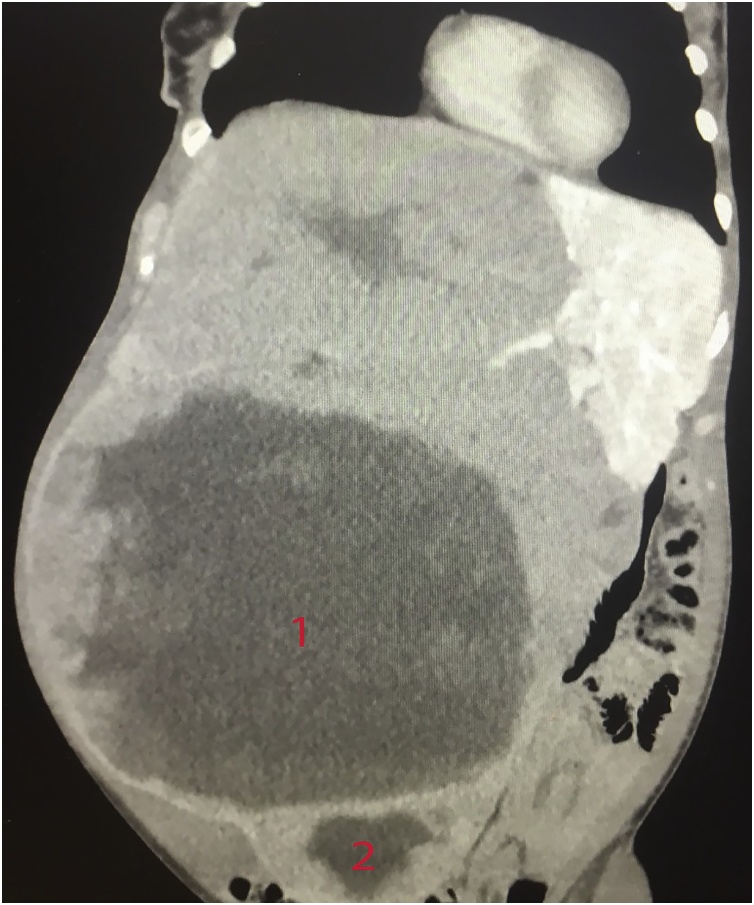
Pre-operative computed tomography scan of liver showing two huge hypodense masses as labeled 1 and 2.

A 46 years old woman was referred to our facility for surgical therapy of an enlarging metastatic gastrointestinal stromal tumour involving the liver. This is on the background of partial gastrectomy for a “benign” tumour in Germany in 1994, which was believed to be the primary. Staging computed tomography scan revealed a grossly enlarged right hepatic lobe secondary to multiple metastases. Two lesions measured 23 cm × 18 cm (oblique axial dimension) and 23 cm × 25 cm × 24 cm (anteroposterior dimension), respectively. The huge tumour led to compression of IVC, right portal and hepatic veins (Fig. 1). The patient’s laboratory studies were within normal except for anaemia (Hb 100). She received neoadjuvant therapy of imatinib, to which the tumour responded with significant size shrinkage.

**Fig. 2:**
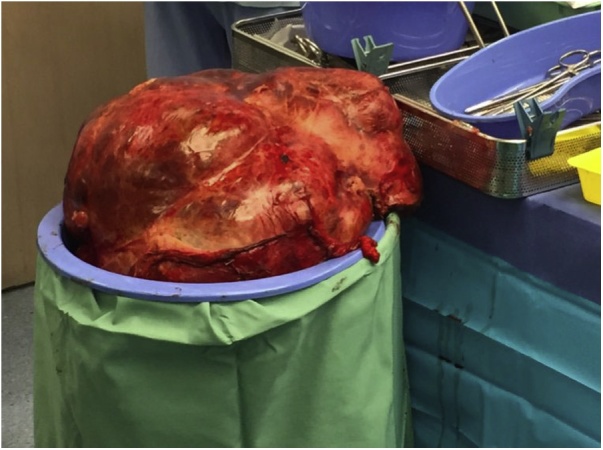
Image showing a grossly enlarged right lobe of liver secondary to metastatic GIST.

She proceeded to undergo an extended right liver resection using cardiopulmonary bypass (CPB) and autotransfusion with intraoperative cell salvage (ICS). Induction of anaesthesia was uncomplicated, followed by placement of lines and a transoesophageal echocardiogram (TOE) probe. On rotational thromboelastometry (ROTEM), maximal clot firmness on FIBTEM was indicative of low fibrinogen (A5 value at 4 mm; A10 value at 4 mm; A20 value at 5 mm). Intraoperatively, a massive tumour of right liver lobe (17 kg) was discovered (Fig. 2). The TOE during early dissection phase was consistent with severe IVC compression and pressure overload on RA/RV suggesting that CPB – instead of veno-venous extracorporeal membrane oxygenation – was necessary to complete the surgery. She was heparinised (20,000 U) to reach activated coagulation time (ACT) of 602 before establishment of CPB. During tumour resection, large volume of fluid and blood products [6 U of packed red blood cells (PRBC), 6 U of fresh frozen plasma (FFP)] was infused to replace intra-abdominal losses. Bleeding from liver edges was controlled adequately with local haemostatic agent (Floseal, Baxter, US). High dose vasopressor support was instituted soon after commencing CPB and early resection – initially with noradrenaline (8 mg/100 ml at 10–50 ml/h) and then vasopressin (2.4 units/h). Eventually, the liver tumour was successfully resected and delivered.

The rates of noradrenaline and vasopressin were running at 50–75 ml/h (16 mg/100 ml) and 6 units/h towards the end of CPB, respectively. Steroids were given and, at this time, there was adequate flow on CPB with no evidence of clot. The patient was then weaned from CBP with good cardiac function. CPB time was 2 h 15 min. A total of 300 mg protamine sulphate was administered for heparin reversal prior to decannulation. Even though her coagulation profile including ACT and ROTEM was acceptable, the haemorrhage was refractory to protamine and blood components. She continued to demand a relatively high dose of vasopressors, although the demand was decreasing over time. Total blood products consumed at that stage were 18 U PRBC, 17 U FFP, 17 U of cryoprecipitate apheresis (equivalent to 34 U of cryoprecipitate derived from whole blood), 3 U pooled platelets and 2500 U prothrombinex. A joint decision among the surgeon, anaesthetist and haematologist was made to administer rFVIIa (90 μ/kg) of rFVIIa (NovoSeven, Novo Nordisk, Denmark) approximately 1-h post-CPB in an attempt to arrest the bleeding. The pH was 6.808 and temperature was 35.3 °C at time of administration.

**Fig. 3:**
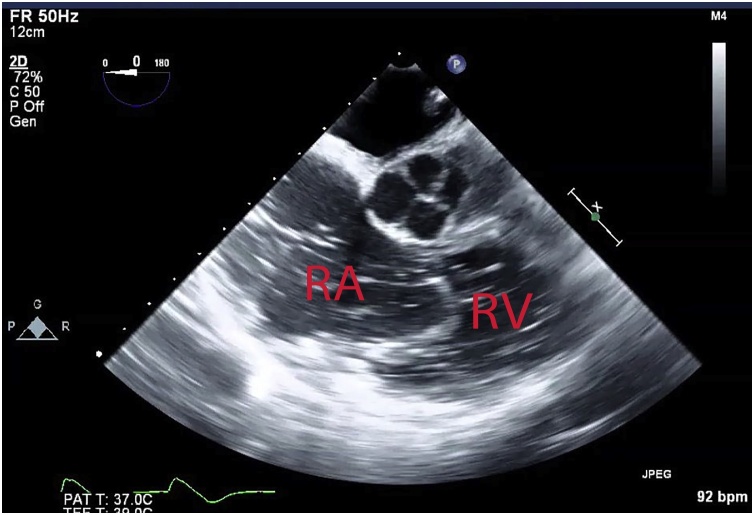
Intraoperative TOE showing extensive thrombi in a markedly dilated right atrium (RA) and right ventricle (RV).

Ten minutes following administration of rFVIIa, the patient became haemodynamically unstable secondary to right ventricular failure and went into cardiac arrest. Intraoperative TOE demonstrated extensive thrombi within the right atrium, right ventricle and bilateral pulmonary arteries (Fig. 3). Heparin was readministered to achieve an ACT of >999 s, CPB was emergently resumed and internal cardiac massage was performed. Right atriotomy and pulmonary atriotomy were attempted successfully to evacuate the aforementioned thrombi. Despite the clearance of right atrium, repeat TOE showed formation of thrombus in left ventricle and aortic arch. There was also noticeable intra-coronary venous thrombosis. The prothrombotic state resulted in eventual propagation of clot into venous drainage line of CPB machine and no flow could be established due to the blocked venous cannulae (SVC and IVC).The treatment was ultimately withdrawn and the patient died soon on the table. Following this event, Therapeutic Goods Aministration (TGA) was notified of the suspected adverse drug reaction.

## 3 Discussion

Intractable intraoperative haemorrhage is a result of both surgical and coagulopathic (nonsurgical) components. Advancement in technology has improved control of bleeding during surgery. Surgical bleeding can be reversed with meticulous diathermy haemostasis. In contrast, nonsurgical bleeding can be corrected with appropriate use of intravenous fluid, blood products and haemostatic agents. However, management of coagulopathic haemorrhage is challenging. Dilutional coagulopathy secondary to excessive fluid and blood products replacement has limited the versatility of replacement therapy in the setting of exsanguination [2]. Its interaction with hypothermia and metabolic acidosis may lead to a downward spiral of clotting capability and eventually death [3].

The need for additional haemostatic agents is apparent with growing interest in the use of rFVIIa. It was initially authorised for administration in patients with haemophilia and alloantibodies to factor VIII and IX. After its success in facilitating haemostasis in a trauma patient less than two decades ago [4], rFVIIa has been increasingly used as an “off-label” rescue treatment in surgery. It aims to rapidly cease refractory bleeding after all conventional approaches have been exhausted [5]. rFVIIa acts pharmacologically via tissue factor-dependent and independent pathway leading to thrombin burst and coagulation [6]. Our patient sustained a rapid blood loss within 30 min (whole blood volume) despite maximal blood products infusion. rFVIIa administration in this setting was conceivable to halt the haemorrhage. Such extreme haemorrhage was likely multifactorial, including haemostatic abnormality secondary to use of anticoagulants and exhaustion of clotting factors and platelets induced by extracorporeal circulation [7].

The rapid development of systemic clotting in our case was unfortunate. The predicament was unclear; however, we suspect that it is likely contributed by various reasons. First, there is an inherent risk of thromboembolism associated with the unlabelled treatment of rFVIIa in surgery [6]. In CPB, upregulation of TF expression may amplify TF-dependent action of rFVIIa, leading to systemic coagulation [8]. A systematic review of all published and unpublished clinical studies estimated thromboembolic events of 1–2% following administration of rFVIIa in severe bleeding [9]. Meanwhile, some authors reported similar complication at a higher rate (9–11%) [[10], [11], [12]]. These studies, though, could not prove a causal relationship between its use and occurrence of thrombotic events. Risk of thrombosis is also higher in disseminated intravascular coagulation [13], which was present in our case as per baseline ROTEM. Other pertinent thrombogenic factors include pre-existing malignancy and systemic inflammatory response syndrome which led to severe vasoplegia requiring high dose vasopressor.

Due to the extensive clotting, we also speculate that the application of topical haemostatic agent in conjunction with ICS could contribute to this. It is well-established that small strands of the haemostatic gel may pass through 40-μm filters of ICS [14]. It may be aspirated into the collection reservoir, potentially leading to systemic circulation of this chemical and subsequent clot formation upon re-infusion [15]. To prevent this, it is recommended to avoid use of cell saver suction while the surgical field is contaminated with topical clotting factors until they have been washed away with 0.9% sodium chloride [16].

## 4 Conclusion

In summary, this case reports on an intraoperative mortality secondary to systemic thrombosis and cardiac arrest involving the use of local haemostatic agent in ICS and administration of rFVIIa. Current evidence, albeit weak, favour the use of rFVIIa in life-threatening haemorrhage, especially in the setting of CPB [12,17]. More randomised controlled trials are needed to validate the efficacy and safety profile of the haemostatic agent. Nevertheless, it is imperative to consider the risk-benefit profile of rFVIIa due to the thrombogenic potential. In our case, provision of rFVIIa to curtail the vicious cycle of coagulopathy was of patient’s best interest as living through the surgery would greatly improve her survival outcome. The authors also feel strongly against the use of local haemostatic gel in conjunction with ICS due to potential systemic circulation of the thrombin.

## Conflicts of interest

No conflict of interest.

## Sources of funding

This research did not receive any specific grant from funding agencies in the public, commercial, or not-for-profit sectors.

## Ethical approval

No ethic is required for this research.

## Consent

Yes it has been documented in the paper.

## Author’s contribution

LSK – main author.

AH and NAA – involved in editing of paper.

DLM – involved in study design and surgery.

## Registration of research studies

This is not a first-in-man case report.

## Guarantor

Lee S Kyang.

Nayef AlZahrani.

David L Morris.

## Provenance and peer review

Not commissioned, externally peer reviewed.
